# Developmental dynamics of Kranz cell transcriptional specificity in maize leaf reveals early onset of C_4_-related processes

**DOI:** 10.1093/jxb/eru152

**Published:** 2014-04-30

**Authors:** S. Lori Tausta, Pinghua Li, Yaqing Si, Neeru Gandotra, Peng Liu, Qi Sun, Thomas P. Brutnell, Timothy Nelson

**Affiliations:** ^1^Department of Molecular, Cellular & Developmental Biology, Yale University, New Haven, CT 06511, USA; ^2^College of Agriculture, Shandong Agricultural University, Taian 271018, China; ^3^Department of Statistics, Iowa State University, Ames, IA 50011, USA; ^4^Institute of Biotechnology, Cornell University, Ithaca NY 14850, USA; ^5^Danforth Plant Science Center, St Louis, MO 63132, USA

**Keywords:** Bundle sheath, C4 photosynthesis, Kranz, leaf development, mesophyll, transcriptome.

## Abstract

The comparison of the cell-specific transcriptomes of bundle sheath (BS) and mesophyll (M) cells from successive developmental stages of maize (*Zea mays*) leaves reveals that the number of genes preferentially transcribed in one cell type or the other varies considerably from the sink–source transition to mature photosynthetic stages. The number of differentially expressed (DE) genes is maximal at a stage well before full maturity, including those that encode key functions for C_4_ photosynthesis. The developmental dynamics of BS/M differential expression can be used to identify candidates for other C_4_-related functions and to simplify the identification of specific pathways members from otherwise complex gene families. A significant portion of the candidates for C_4_-related transcription factors identified with this developmental DE strategy overlap with those identified in studies using alternative strategies, thus providing independent support for their potential importance.

## Summary

The measured differential expression of genes between bundle sheath and mesophyll cells at successive developmental stages of the maize leaf is used to identify C_4_-photosynthesis-related candidates.

## Introduction

C_4_ photosynthesis has evolved independently in many plant groups, both monocot and dicot (Sage *et al.*, 2011; Aliscioni *et al.*, 2012; Denton *et al.*, 2013). The majority of existing C_4_ species rely on the metabolic cooperation of leaf bundle sheath (BS) and mesophyll (M) cells with a Kranz-type anatomy surrounding the venation (Langdale, 2011). In this two-cell metabolism, M cells perform primary carbon assimilation and adjacent BS cells perform a linked primary carbon reduction. M cells fix atmospheric CO_2_ into C_4_ acids, which are passed through plasmodesmata to adjacent BS cells and decarboxylated. The BS is specialized with a permeability boundary to retain the released CO_2_ and to exclude O_2_, thereby enhancing the Rubisco-initiated carbon reduction steps (Nelson, 2010). BS cells seem to be developmentally related to the endodermal cells that surround the vasculature of the root and stem (Slewinski, 2013).

Many studies have evaluated the regulation of individual genes whose products are BS- or M-specific, and several studies have compared the transcriptomes of mature BS and M cells (Hibberd and Covshoff, 2010; Langdale, 2011). However, mature tissues provide limited insight into the developmental mechanisms that produce mature BS and M cells. Whole-leaf transcriptome and proteome studies of sequential developmental stages from the base to tip of the young maize leaf (Nelson, 2011) revealed a developmentally dynamic process in the appearance and disappearance of transcripts, transcription factors and other proteins, with a significant portion reduced to low level or absent in mature tissue (Majeran *et al.*, 2010; Li *et al.*, 2010; Pick *et al.*, 2011). This suggests that some important factors may appear and act at early stages only.

In this study we compare the transcriptomes of maize BS and M cells at three successive developmental stages: sink–source transition (SST), maturing, and mature. In addition to resolving the timing of processes during the differentiation of the two cell types, the dynamics of differential BS/M expression of genes can be used as a filter for identifying new candidates for C_4_-related genes. It also provides a means to select pathway candidates among gene family members. We compare candidate C_4_-related genes identified by our BS/M laser microdissection (LM)-based strategy with the candidates identified by alternative strategies. Consensus candidates identified by multiple methods warrant further study.

## Materials and methods

### Cell and RNA isolation

Maize Mo17 and B73 leaf sections were collected from synchronously grown plants and preserved in 100% ice-cold acetone as described in Li *et al.*, 2010. After fixation in paraffin, BS and M cells were captured using a PixCell IIe or Veritas LM system (Life Technologies) and the RNA recovered with a Picopure RNA isolation kit (Life Technologies) according the to the manufacturer’s instructions. A sample of 50–100ng of total RNA was amplified through one round using the RiboAmp HS plus RNA amplification kit (Life Technologies). RNA quality was monitored using a Bioanalyzer 2100 (Agilent).

### Library preparation and Illumina sequence analysis

Library construction, Illumina sequencing, and sequence analysis were done as previously described (Li *et al.*, 2010). The background level was estimated by calculating the alignment density in genomic regions that are at least 10kb away from any exon. Based on the estimated background level and the Poisson model, we calculated the *P*-value for testing whether this gene is expressed or not (whether the observed counts arose from background noise or they were true signal) by calculating the probability of observing the observed counts or more counts if all reads mapped to this gene were background noise. We controlled the false discovery rate (FDR) at 0.1% using Benjamini and Hochberg’s procedure (Benjamini and Hochberg, 1995). Raw datasets are available at NCBI Gene Expression Omnibus (www.ncbi.nim.nih.gov/geo), accession GSE 54272.

DESeq (Anders and Huber, 2010), an R package, was used with upper quartile normalization method (Bullard *et al.*, 2010) to test for differential expression between BS and M cells in each stage. The Benjamini and Hochberg method was applied to the list of resulting *P*-values to control FDR (Benjamini and Hochberg, 1995). Genes that were differentially expressed between BS and M cells (FDR<0.01) were then separated into BS or M expression using log_2_-fold change of BS/M. The functional annotation of identified genes was based on the Mapman pathways (http://mapman.gabipd.org/web/guest/mapmanstore—Zm_B73_5b_FGS_cds_2012download) with manual corrections and further refinement using Putative Orthologous Groups (http://cas-pogs.uoregon.edu/#/) (Walker *et al.*, 2007; Tomcal *et al.*, 2013) and Phytozome (http://www.phytozome.net/). Protein data were compared using the Plant Proteome Database- PPDB (http://ppdb.tc.cornell.edu/) (Sun *et al.*, 2009). Homeologue identification was performed using data provided by http://skraelingmountain.com/datasets.php (Schnable *et al.*, 2012).

For comparison of identified transcription factors from other reports, all gene names were checked with B73 RefGen_v2 to ensure that the maize identifications were consistent.

## Results

### The expression of many genes differentially expressed between BS and M cells peaks and declines before maturity during C_4_ leaf development.

We inventoried the transcripts present in BS and mesophyll M cells at three sites along the developmental gradient of a nine-day-old 3rd maize leaf, using LM and RNA-seq, as described in Li *et al.* (2010). The three sites corresponded to SST, maturing, and mature developmental stages, located at –1cm, +4cm and +9cm (leaf tip), relative to the completion of the sink–source transition, which we previously showed is located at the point touched by the ligule of the 2nd leaf (Li *et al.*, 2010; Majeran *et al.*, 2010). The SST is exposed to a lower level of light than the two succeeding stages.

**Fig. 1.**
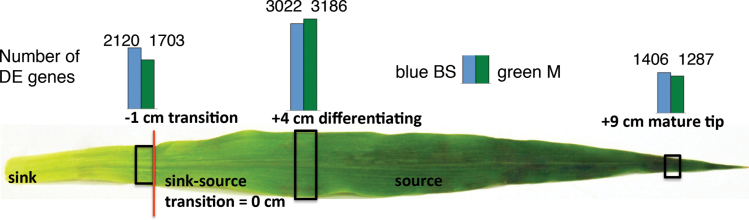
Developmental dynamics of BS/M differential expression (DE). Three sections from the third leaf of a 9-day-old maize plant representing locations of sink–source transition (SST), maturing, and mature developmental stages, the source material for BS and M LM capture. Details of sample site location, anatomy and ultrastructure are in Li *et al.* (2010) and Majeran *et al.* (2010). Blue bars above the leaf represent the number of DE genes found to be BS-biased and green bars represent the number of M-biased DE genes.

Expressed genes above background were determined as described in materials and methods; false discovery rate (FDR) was controlled at 0.1%, 27 819 genes were expressed, based on the filtered gene set of maize assembly v2. For each stage, the BS- and M-differentially expressed (DE) genes were identified by comparing the transcript levels between BS and M cells at each stage, using the R package, DESeq, as described in materials and methods. This analysis identified 7994 genes that were either BS- or M-enriched in expression (Supplementary dataset S1). In this report, DE is defined as those genes whose transcripts were significantly higher in one cell type versus the other as determined by DESeq. BS- or M-enriched (or –biased) genes are defined as those whose transcripts were more abundant in that cell type versus the other; “-specific” genes are defined as those that were exclusively detected in one type. The majority of DE genes (~85%) ranged from 2-fold enriched to completely specific for BS or M. The number of genes with cell-enriched transcripts was greatest during the maturing stage, and was markedly less in the mature stage (Fig. 1). Transcripts of 1703, 3186, and 1287 genes were M-cell-enriched in the SST, maturing, and mature stages, respectively, a total of 4154 unique genes that were enriched in M cells in at least one stage. Transcripts of 2120, 3022, and 1406 were BS-enriched, a total of 4043 unique genes that were enriched in BS cells in at least one stage.

Most DE genes for BS and M cells are not absolutely cell-specific, reinforcing observations in many organisms that cell-specific traits are produced through a combination of transcriptional and translational mechanisms (e.g. Ramskold *et al.*, 2009). The C_4_ carbon shuttle genes, all transcribed at a high level, exhibit a range of BS-M transcriptional ratios in the three developmental stages. Comparisons of BS and M transcriptomes isolated from a single stage by the same LM method reveal a broad range of ratios of differential expression for different genes, suggesting that the BS-M distributions are not a cross-contamination artefact. It is also consistent with the distributions observed in proteomic measurements performed on mechanically separated cells from the same sources (Friso *et al.*, 2010; Majeran *et al.*, 2010). As shown previously, there is good correlation between the BS/M transcript ratio and the protein BS/M ratio of photosynthesis-related genes (Li *et al.*, 2010).

### C_4_-associated metabolic genes are BS/M-cell-specific from initial expression near leaf sink–source transition, peak in level at midblade, decline at mature tip

**Fig. 2.**
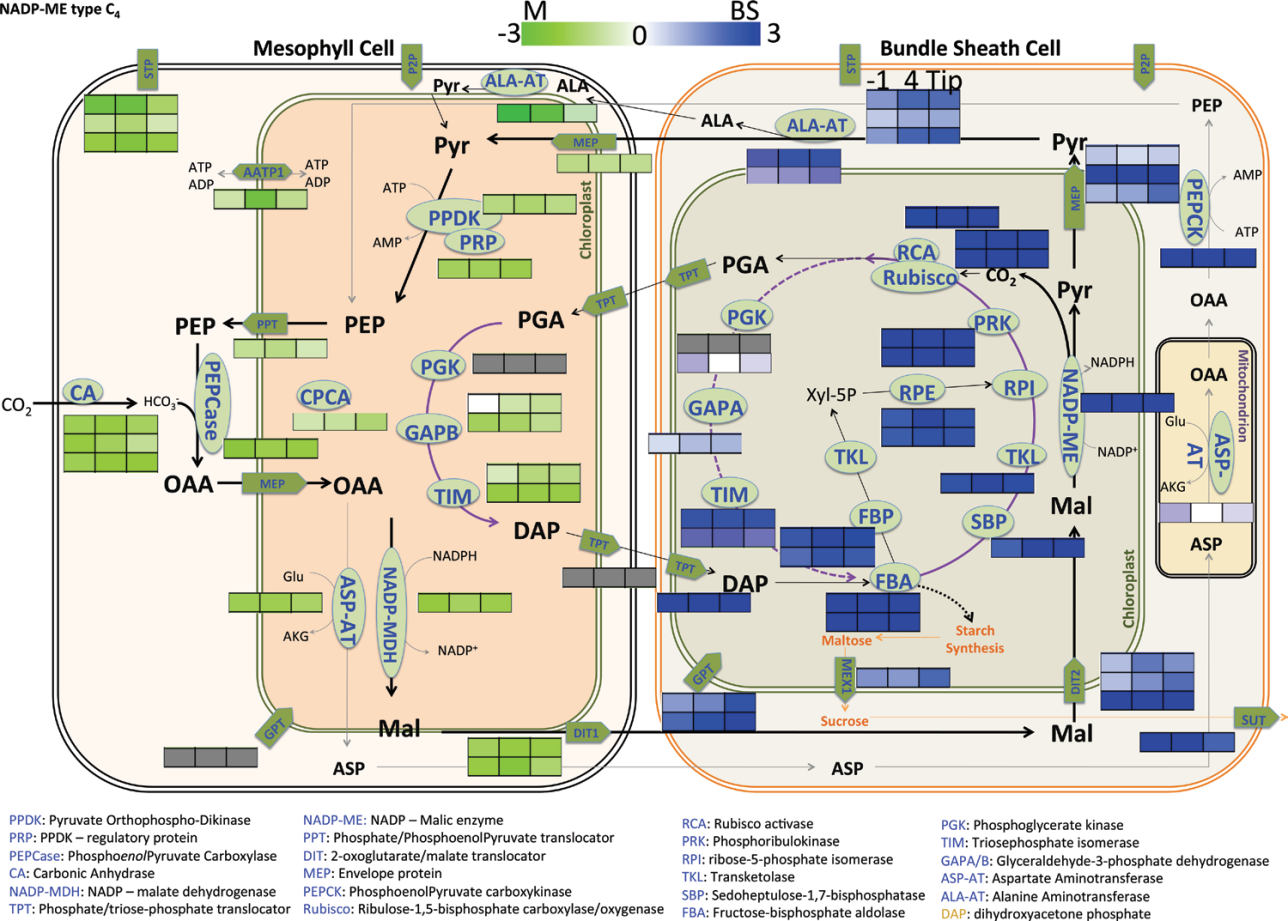
C_4_ cycle genes required for cell cooperation are cell-biased at SST. The C_4_ cycle between BS and M cells relies on numerous enzymes expressed in either cell type. Heat maps of three boxes next to enzyme names represent the log_2_-transformed BS/M expression ratio at the three developmental stages sampled (SST, maturing, mature). Each row indicates the results from a gene with the same proposed activity. Green signifies an M-biased ratio and blue represents a BS-biased ratio. Modified from Li *et al.*, 2010 and detailed expression data is found in Supplementary dataset 2.

Genes encoding proteins known to be either BS- or M-specific in mature leaves exhibit a transcriptional bias at the first stage sampled, the SST stage, where Kranz anatomy is evident, but cells have not yet transitioned to a photosynthetically active state (Majeran *et al.*, 2010). For instance, transcripts for the core C_4_ pathway enzymes are all at least two-fold more highly expressed in one cell type versus the other in the SST stage, which is surrounded and shaded by an older leaf (e.g. log_2_ BS/M for MDH=–2, PepC=–2.2, NADP-ME=3.1, TKL= 2.6) (Fig. 2 and Li *et al.*, 2010). This pattern is also true for cell-type-biased genes in photorespiration, starch and sucrose metabolism, sulfur assimilation, and other pathways that become active following the SST (Supplementary Fig. S1). In the subsequent well-illuminated stages (maturing and mature), the level of expression increases, but the cell type bias is maintained.

Many DE transcripts encoding products that function in mature leaves peak in abundance at maturing stage before declining significantly at mature stage. We do not present here a K-means clustering of DE genes by their expression dynamics over the three stages, as the peak-and-decline developmental dynamic was previously documented for individual genes at the whole-leaf transcriptome level (Li *et al.*, 2010; Pick *et al.*, 2011). The abundance of most of the corresponding proteins does not follow this pattern. Instead, C_4_ photosynthesis-associated proteins and other metabolic pathway proteins generally remain abundant at the tip of the leaf, without the decline observed for their transcripts (Friso *et al.*, 2010; Majeran *et al.*, 2010).

### Members of the same gene family are differentially expressed in BS and M cells

**Fig. 3.**
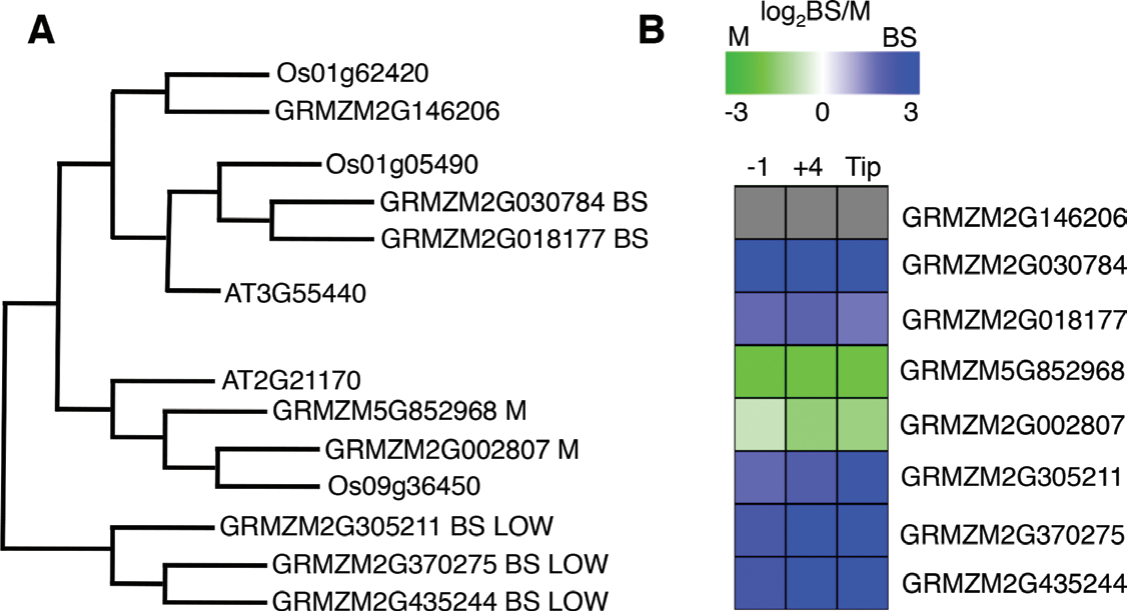
TIM family members assigned to BS or M. (A) TIM family putative orthologue group, POG, represented by phenetic tree generated from protein MUSCLE alignment and using UPGMA (unweighted pair group method and arithmetic mean) (Edgar, 2004; Tomcal *et al.*, 2013). Members with similar cell-biased expression cluster together. (B) DE heat map of TIM genes. Grey boxes for the cytosolic form of TIM represent no DE data. Green signifies an M-biased ratio and blue represents a BS-biased ratio based on the log_2_-transformed BS/M ratio for each section. Detailed expression data is found in Supplementary dataset 2.

Cell expression ratios and cell-specific co-expression patterns can be used as filters to infer the BS/M-relevant member(s) of otherwise redundant gene families annotated with a general function. For example, some gene families that encode activities found in both BS and M cells seem to have subfunctionalized, with distinct members driving expression in each cell type (e.g. triosephosphate isomerase, TIM), whereas others use the same members in both cells (e.g. PGK) (Figs 2 and 3). In the case of TIM, a gene family encodes activities for steps in glycolysis and also in the production of d-glyceraldehyde 3-phosphate (DAP), a Calvin cycle intermediate, in both BS and M cells. Two closely related *TIM* genes (GRMZM2G002807 and GRMZM5G852968) are highly expressed in M cells and are maize homeologues (Schnable *et al.*, 2011). The other *TIM* genes have a more complex relationship. Of the five BS DE genes in the same POG (putative orthologue group; Walker *et al.*, 2007; Tomcal *et al.*, 2013), two are highly expressed (GRMZM2G030784, GRMZM2G018177) and three are expressed at a much lower level (~10× less) but still with a BS-enhanced ratio (GRMZM2G370275, GRMZM2G435244, GRMZM2G305211). The two more highly expressed BS-enriched *TIMs* are also homeologues, but the three less expressed genes are not. Transcript amounts from a sixth gene, encoding the cytosolic form of TIM (GRMZM2G146206) are high at the leaf base (all-cell sample) but low in BS and M cells of subsequent stages. The cytosolic protein and its transcripts are both BS- biased. Generally, the similarity of expression patterns among gene family members can be predicted by their sequence similarity dendrogram (Fig. 3A, Supplementary dataset S2). The divergence of expression among such closely related gene family members suggests that comparison of their *cis*-regulatory regions may reveal motifs for cell-specific expression.

Additional examples of gene families that contain both BS and M-enriched family members were found in metabolic pathways both related and unrelated to C_4_ photosynthesis. For example, five closely related pyruvate kinase genes exhibit distinct expression patterns in the leaf, including two M-biased, two BS-biased, and one BS-M unbiased. Of the five cytosolic glutamine synthetases (GS1), transcripts for two are M-enriched and three are BS-enriched (Cren and Hirel, 1999). The presence of GS activity in both BS and M cells has been documented (Becker *et al.*, 2000) and the transcript patterns correspond well with protein location and relative abundance (Martin *et al.*, 2006; Sun *et al.*, 2009). In contrast, a single DE glutamate dehydrogenase gene (*gdh1*) is BS-expressed and the corresponding protein is found only in the BS (Friso *et al.*, 2010; Majeran *et al.*, 2010; Supplementary dataset S2).

### Use of developmental and cell-type co-expression to infer gene function and interactions

The developmental dynamics of cell-type specificity can permit the assignment of putative functions of members of large families before direct functional tests. As an example of the inference of function, our data supports the current understanding of an additional decarboxylation cycle involving phosphoenolpyruvate carboxykinase (PEPCK), alanine aminotransferase (AlaAT) and aspartate aminotransferase (AspAT) (Leegood and Walker, 2003; Pick *et al.*, 2011). *PEPCK* (GRMZM2G001696) was found to be highly expressed in BS in all three sections with the highest expression at the tip, which is unusual for a C_4_ gene (Fig. 2, Supplementary dataset S2). The DE genes include one AspAT isoform expressed in M (GRMZM5G836910) but not the one (GRMZM2G094712) detected by another study in mature BS cells (Chang *et al.*, 2012). We find GRMZM2G094712 is BS-enriched only at SST, the stage at which it is expressed at the highest level, and similar to the AspAT (GRMZM2G033799) that was DE in this experiment. Neither of the BS AspATs have high transcript accumulation profiles in this experiment, but both proteins have been identified in BS cells (Friso *et al.*, 2010; Majeran *et al.*, 2010), suggesting regulation is post-transcriptional. Among the large AlaAT gene family, at least five genes meet our DE criteria. One M-biased member (GRMZM2G088064) exhibits the dynamic pattern of C_4_ genes, starting at SST, peaking at maturing stage, and declining at mature. The corresponding protein is found in field-grown leaves, but was not identified in the proteome of M cells (Friso *et al.*, 2010; Majeran *et al.*, 2010). Of the remaining AlaAT activities, transcripts for all are BS-enriched; three were found in the proteome of BS cells (Friso *et al.*, 2010; Majeran *et al.*, 2010). Two AlaAT genes (GRMZM2G053999, GRMZM5G840582) exhibit the levels and developmental patterns of C_4_-related genes.

As another example of the inference of gene function based on cell-specific expression pattern, aquaporins constitute large families of transporters with many leaf-expressed members. Aquaporins were initially associated with water homeostasis, but some are involved in transport of solutes such as CO_2_ (Uehlein *et al.*, 2003; Hanba *et al.*, 2004). Water transport is crucial for photosynthesis and phloem loading. In the maize leaf, BS cells are surrounded by a suberin boundary similar to the Casparian strip that surrounds root endodermal cells and makes the movement of water aquaporin-dependent (Amodeo *et al.*, 1999; Johansson *et al.*, 2000). The aquaporin family in maize has over 30 members in four major subfamilies that play important roles in the leaf (Chaumont *et al.*, 2001; Johanson *et al.*, 2001; Heinen *et al.*, 2009).

We observe 16 BS-biased and 4 M-biased aquaporin family genes, of which a subset is expressed at high levels beginning at SST, consistent with the building of infrastructure for subsequent C_4_ processes (Supplementary dataset S2). Members of the *PIP* (plasma membrane intrinsic protein) subfamily were the most abundant in either cell type, although all four major subfamilies were represented in the BS DE list. The BS-enriched DE genes included seven PIP members. The M-enriched DE genes included four *PIPs* with differing dynamic patterns, only one of which was M-enriched at all developmental stages. Six BS and two M-biased *PIPs* are closely related by sequence but were not found to be maize homeologues. The two M-enriched DE *PIPs* (GRMZM2G154628, GRMZM2G081192) are in the same sub-cluster and both show a pattern of highest expression at the SST stage. The BS-M biases we observe for transcripts for the 21 aquaporins agree with those found in mature stage in another study (Chang *et al.*, 2012), except for two for which we find a lower BS/M ratio at mature stage, and three for which we observe a differing specificity at an earlier stage. Transcripts for PIPs are abundant at the leaf base (Li *et al.*, 2010) where PIPs are likely to be incorporated into cell walls during cell division; the SST cell-type-enhanced accumulation we observe may be a continuation of this process.

Cell-specific co-expression information that includes early stages can help to associate cognate pairs of interacting proteins among multiple candidates in gene families, even if expression levels are low. For instance, Aux/IAA repressors interact with auxin response factor (ARF) transcription factors in response to auxin (Hagen and Guilfoyle, 2002). Both partners in this interaction come from gene families, making possible many theoretical pairings. A cell-specific co-expression strategy was previously employed in *Arabidopsis* to identify candidate pairings (Gandotra *et al.*, 2013). Our cell-type co-expression data considerably limits the candidate partners in BS and M cells. For example, in M cells, one ARF and six Aux/IAAs are preferentially expressed at the SST stage (Supplementary dataset S2). The ARF and three Aux/IAAs continue to show M expression through the mature stage at the tip of the leaf. In BS cells, three ARFs and two Aux/IAAs are preferentially expressed at the SST stage and only one ARF and one Aux/IAA remain BS-expressed out to the mature tip.

### The most highly expressed DE transcripts are abundant at all stages

**Fig. 4.**
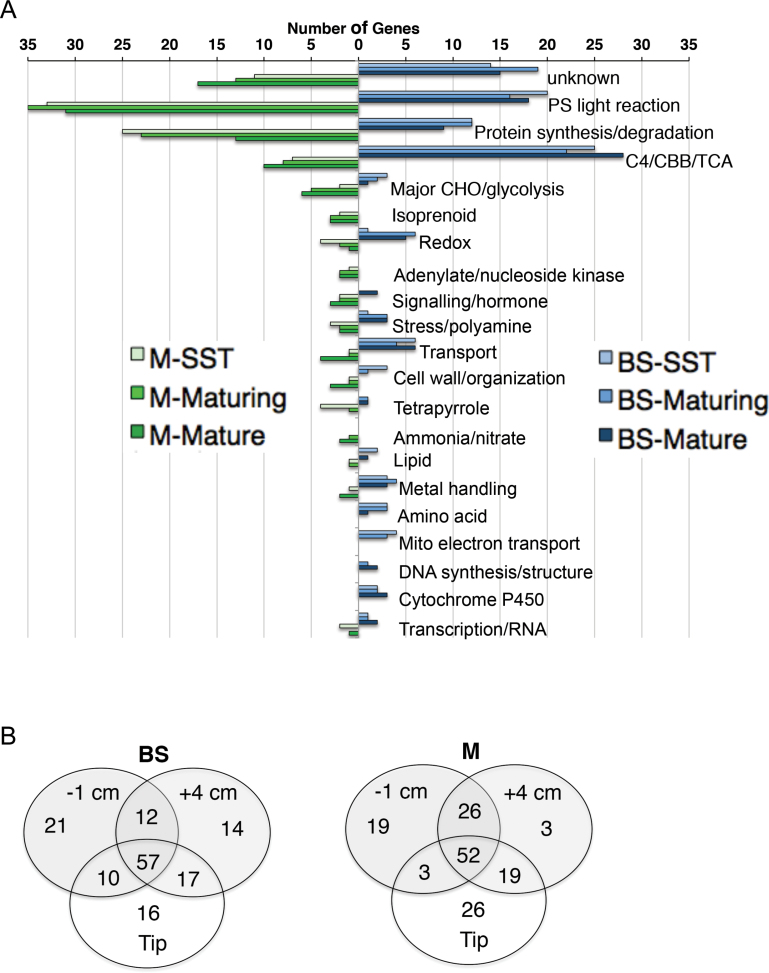
Most highly expressed DE genes present throughout blade. (A) The BS or M DE lists were filtered for the top 100 most highly expressed DE genes in each of the three developmental stages. For each section, genes were placed in Mapman categories and plotted by number of members. The categories with the most members were as expected and remained high throughout in both cell types. (B) Venn diagram of genes in the top-100 DE set for each developmental stage. Many genes were found in top-100 of all three stages. The top-100 set of maturing stage shared the most with those of the other two stages. In this set, only 14% of BS genes and 3% of M genes were maturing stage-unique. In contrast, 19% of M genes in SST and 26% of M genes in the mature stage top-100 sets were stage-unique.

As transcripts for C_4_ enzymes are all expressed at a high level throughout the green blade, it is likely that genes encoding supportive functions such as transporters might also be distinguished by their high transcript accumulation profiles. For example, in maturing stage, we detect C_4_ NADP-malic enzyme (ME) with a normalized expression value of 1178 and C_3_ NADP-ME with value of 3. Among BS- or M-enhanced transcripts, we compared the functional categories of the 100 most highly expressed genes at each stage (Fig. 4A). Note that long transcripts are not over-represented in this analysis because the LM-RNA-seq strategy includes an RNA amplification step that biases reads to the 3’ ends of transcripts. As for the major C_4_ enzymes, cell-type-enriched gene expression is apparent in the SST stage. The majority of highly expressed genes remain in the top-100 class throughout the three stages (Fig. 4A and Supplementary dataset S3). As expected, most are photosynthesis-associated, but protein synthesis and degradation and redox are also represented. Several transporters are highly expressed at all stages, including two aquaporins and two mitochondrial membrane transporters (DiT2 and AAC) all of which are BS-enriched. The C_4_-related DiT1 transporter is M-enhanced and highly expressed in all stages (Taniguchi *et al.*, 2004).

Several of the highest cell-enriched transcripts are associated with activities proposed to support C_4_ photosynthesis. Transcripts for the subunits of the NAD(P)H dehydrogenase (NDH) complex are notably high in BS cells at all three stages, consistent with the high protein levels previously observed in BS chloroplasts (Darie *et al.*, 2006; Majeran *et al.*, 2008). The corresponding activity is proposed to be important for preventing CO_2_ leakage from the BS when ME decarboxylation is faster than Rubisco carboxylation (Takabayashi *et al.*, 2005). M cells accumulate high levels of transcripts for isoprenoid and tetrapyrrole-pathway activities that produce chlorophyll and other structural components for photosynthesis and respiration. Many of the highly expressed and cell-biased transcripts encoding currently unknown activities or structures may be additional components that support C_4_ capacity.

A subset of BS-M differentially expressed (DE) genes were only among the top-100 most highly expressed for a single stage. In M cells, transcripts from 19 DE genes accumulate to very high levels only in the SST stage (Fig. 4B). Three are highly abundant at maturing stage only and 26 at mature stage only. In BS cells, 21, 14, and 16 are at very high levels in SST, maturing, and mature stages only. Although found to be differentially expressed, most of these very highly expressed genes are not absolutely cell-specific. Proteomic data from the mature stage (Friso *et al.*, 2010; Majeran *et al.*, 2010) provides evidence for 69% of the corresponding predicted proteins in BS cells and 47% in M cells, with distribution ratios consistent with the transcript ratios.

Those very highly expressed only at SST stage include BS-enriched genes involved in lipid synthesis, a plastid development protein, and chloroplast chaperonins, and M-enriched genes for four ribosomal proteins, two tetrapyrrole synthesis proteins, and other chloroplast-related proteins, all presumably associated with building chloroplast capacity. In contrast, the M-enriched transcripts that were in the top-100 set only at mature stage are chloroplast-related, but seem to be dedicated to regulation or protection. Among these are transcripts for fibrillin, a plastoglobule protein thought to protect against stress (Singh and McNellis, 2011); peroxiredoxin, an antioxidant enzyme; and pyruvate phosphate dikinase (PPDK) regulatory protein, which controls PPDK activity by phosphorylation (Burnell and Chastain, 2006; Chastain *et al.*, 2008; Friso *et al.*, 2010). BS-enriched transcripts at this stage include genes associated with energy capture (H(+)-ATPase, GPT2 transporter), photorespiration (glycine cleavage H protein) and sulfate transport.

### Stage-enhanced DE transcripts: SST stage includes putative BS secondary wall cellulose synthase; mature stage-enhanced DE genes are enriched in stress-related classes

**Fig. 5.**
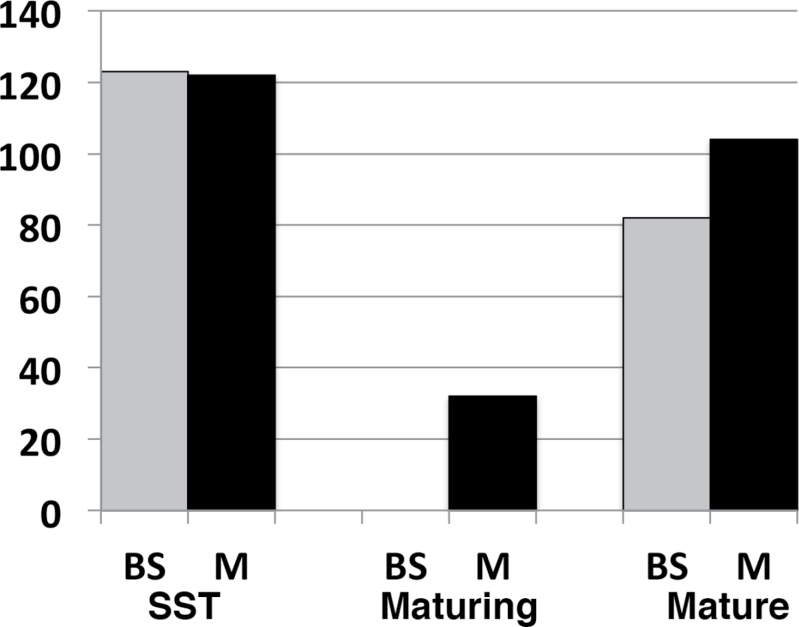
DE genes expressed in only one stage. DE genes that were expressed in only one of the three stages are plotted for each cell type. The number is highest for the SST stage and smallest for maturing stage for both cell types.

The SST and mature stages exhibited notable examples of DE transcripts that are very low or nearly absent at the other two stages; the maturing stage exhibited few such examples (Fig. 5). Gene lists were generated such that the normalized expression value of less than 0.2 in the other two sections was considered non-expressed. For most of these stage-enhanced genes, the level of expression is much less than that for C4 pathway genes (Supplementary dataset S4). The following are highlights for the two stages:

#### Sink–source transition stage

**Fig. 6.**
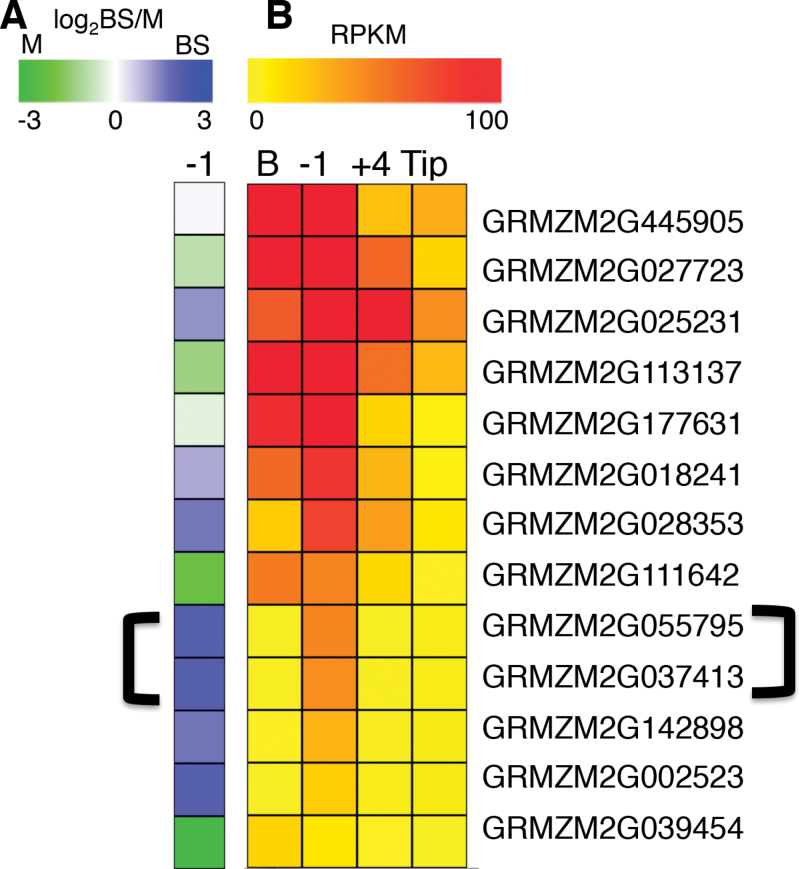
Cellulose synthase genes expressed in BS at SST stage. (A) A heat map representing the log_2_ BS/M ratio for a family of related *CesA* homologues where blue represents BS and green M. Two BS expressed members indicated by a bracket were found only expressed in the SST stage. (B) Heat map from low (yellow) to high (red) represents the whole section RNA-seq data (Li *et al.*, 2010) for the same genes. Note that the two bracketed BS genes are also only present in the SST stage.

The SST stage-enhanced transcripts include BS-specific sucrose synthase, consistent with the prevailing sink metabolism, and cellulose synthase (CES), perhaps associated with strengthening of BS physical properties. According to corresponding proteomic data from the same source material (Majeran *et al.*, 2010), levels of this sucrose synthase protein decline from (earlier stage) leaf base to SST and subsequent stages. The SST stage uniquely exhibits transcripts from numerous secondary wall synthesis-associated genes. Among seven cell wall-related BS-enhanced genes are two maize *CES* genes closely related to each other and to the *CESA8/IRX1 Arabidopsis* gene, which causes enhanced drought and osmotic stress tolerance when mutant (Turner and Somerville, 1997; Taylor *et al.*, 2003). Secondary cell walls are laid down following cell expansion to enhance mechanical strength (Endler and Persson, 2011). All but one (GRMZM2G039454) of the many maize *CES* homologues in the *CES* POG are most highly expressed in the SST stage (Fig. 6). The most highly expressed (GRMZM2G445905) is not DE, but equal in both cell types. Also consistent with this SST-localized and BS-specific developmental pattern are transcripts for the maize homologue of the secondary wall master regulator NST1 (GRMZM2G171395), three lignin biosynthetic genes and a laccase, suggesting that a burst of BS-specific wall strengthening occurs immediately before blade emergence (Mitsuda *et al.*, 2007; Berthet *et al.*, 2011; Wang and Dixon, 2012). Also sharing this SST-localized, BS-specific pattern are three RALFs (rapid alkalinization factors) generally associated with growth inhibition (Bedinger *et al.*, 2010), and here perhaps terminating cell expansion for BS cells before the source transition.

Several other sink-related genes are expressed in the SST stage and decline in subsequent stages. These include invertase 2 (*IVR2*, GRMZM2G089836, M-enriched), which is active in sink tissues (Kim *et al.*, 2000), and *ZmABI39* (GRMZM2G172621, BS/M equal) a member of the ABI3-VP1 family that is in the same orthologue group as the *Arabidopsis* gene *HSI2*, a sink tissue repressor of seed maturation genes (Tsukagoshi *et al.*, 2005). Several amino acid transporters serve sink tissue in *Arabidopsis* (Su *et al.*, 2004; Hammes *et al.*, 2006). In maize, one amino acid transporter candidate (GRMZM2G404888, BS-biased at SST) is expressed most highly in the leaf base and SST stages (Li *et al.*, 2010; Supplementary dataset S2).

A sink-tissue-associated calcium-dependent kinase family (CPDK, CPK) is important in stress response (Ludwig *et al.*, 2004). In maize, 40 CPKs are identified (Kong *et al.*, 2013), four of which (*ZmCPK5, 23, 29, 30*) show expression levels highest in the leaf base and decrease in subsequent stages (Li *et al.*, 2010; Sekhon *et al.*, 2011). We observe that *ZmCPK29* and *ZmCPK30* (homeologues) are M-enhanced at SST, *ZmCPK23* is BS-enhanced, and *ZmCPK5* is uniformly expressed (Supplementary dataset S2).

#### Mature stage

The mature stage seems to undergo a decline in photosynthesis-related transcripts and a rise in stress-related transcripts, as has been noted in two previous transcriptome studies (Li *et al.*, 2010; Pick *et al.*, 2011). We observe 104 genes that are mature- and M-enhanced and 82 genes that are mature- and BS-enhanced. The M-enhanced list is rich in maize genes in POGs associated with stress responses in *Arabidopsis*. For example, the maize gene GRMZM2G066870 corresponds to *Arabidopsis* AT3G05880 (RARE-COLD-INDUCIBLE 2A) a gene induced by low temperatures, dehydration, salt stress, and ABA (abscisic acid) (Capel *et al.*, 1997; Nylander *et al.*, 2001). Of the five transcription factors included in the M-specific group, four have *Arabidopsis* homologues that are induced by stress conditions including H_2_O_2_ (At1g10585), jasmonic acid (JA; AtWRKY50-At5g26170; Gao *et al.*, 2011), salt and other abiotic and biotic stresses (*AtWRKY33-At2g38470*; Birkenbihl *et al.*, 2012; Maekawa *et al.*, 2012), carbon/nitrogen status, and various abiotic stresses (*bHLH92- At5g43650*; Jiang *et al.*, 2009). Also in the M-specific list are a raffinose synthase gene potentially related to stress (Nishizawa *et al.*, 2008) and several ARD genes likely to act in a stress-related methionine salvage pathway, as described below in the Discussion. The BS-specific list includes a smaller number of genes potentially related to stress.

### Differential expression of C_4_-related genes

DE patterns in BS and M cells beginning at an early stage may be a common feature of genes with significant roles in C_4_ metabolism and Kranz anatomy, as shown for the well-characterized C_4_ pathway enzymes. The following are examples in support of this hypothesis. The TFs Golden2 (*G2*, GRMZM2G087804) and Golden Like1 (*GLK1*, GRMZM2G026833) influence BS/M chloroplast specialization, and are cell-enriched (*G2* in BS, *GLK1* in M; Hall *et al.*, 1998; Rossini *et al.*, 2001). Both *ZmG2* and *ZmGLK1* are expressed in the leaf base, but peak at the mature stage (Li *et al.*, 2010). Two transcriptome studies (Li *et al.*, 2010; Chang *et al.*, 2012) confirm the DE patterns for mature stage BS and M cells. We find that these patterns are present at all three developmental stages, as expected for regulators of cell specification (log_2_ BS/M G2: 0.93, 2.36, 1.76; GLK1: –1.8, –1.4, –1.7). Another example, the mitochondrial transporter A BOUT DE SOUFFLE (BOU) influences photorespiration in *Arabidopsis* (Eisenhut *et al.*, 2013), and the maize homologue is likely to be C_4_-related. We find that *ZmBOU* (GRMZM2G024823) is expressed robustly in a highly cell-biased fashion at all three stages, from SST to mature (log_2_ BS/M, 1.6, 3.2, 2.8).

The RBCL RNA S1-Binding domain protein (*RLSB*, GRMZM2G087628), binds the mRNA of the large subunit of Rubisco (*rbcL*) and influences *rbcL* expression (Bowman *et al.*, 2013). Although RLSB protein is detected only in BS chloroplasts, a transcriptome study detected the presence of transcript in M cells (Chang *et al.*, 2012). We find this pattern is developmentally dynamic: the SST stage exhibits a BS-bias (log_2_ BS/M 0.3), maturing stage an M-bias (log_2_ BS/M –1.3), and mature stage a slight M-bias (log_2_ BS/M –0.2). Possibly consistent with this, a transgenic knockdown of the *RLSB* transcript in maize had a large effect early on, but fades as the leaf matures (Bowman *et al.*, 2013).

As a final example of a C_4_-related DE pattern, the maize Scarecrow TF homologue *ZmSCR* was shown recently to be critical for Kranz patterning of BS and M cells (Slewinski *et al.*, 2012). We observe that *ZmSCR* (GRMZM2G131516) is significantly BS-biased at all three developmental stages sampled (log_2_ BS/M 0.4, 0.7, 0.9); it is particularly highly expressed in the leaf base (Li *et al.*, 2010).

The consistently BS/M-biased patterns of expression of these and other C_4_-related genes suggests that further interrogation of our data set based on DE patterns can be used to infer other likely candidates with roles in Kranz anatomy and C_4_ metabolism

### BS/M specificity as a filter for TF candidates with C_4_ roles

**Fig. 7.**
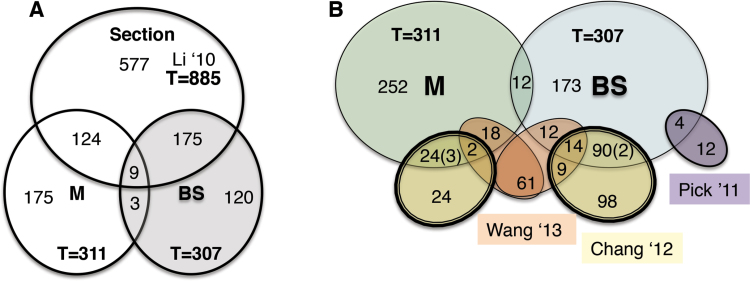
Comparing strategies for transcription factor identification. (A) Comparison of BS and M DE-identified TFs with those previously identified from the whole sectional data, after elimination of any genes not found in B73 RefGen_v2 (Li *et al.*, 2010). More BS transcription factors overlap than M. The overlap between the BS and M represents expression patterns that switched DE cell-bias over the developmental gradient. (B) Comparison of BS and M DE-identified transcription factors with other published C_4_ transcription factor lists. Yellow ovals represent data from Chang *et al.*, 2012; orange ovals represent data from Wang *et al.*, 2013; and purple oval represents data from Pick *et al.*, 2011. In the overlap between the Chang *et al.*, 2012 data and the LM data (this study) the numbers in parenthesis represent expression patterns that changed cell-bias over the developmental gradient and thus were not restricted to the cell type identified by cell separation.

Previously, 938 genes annotated as transcription factors (TFs) were identified as expressed at some stage during leaf development (Li *et al.*, 2010). To compare this list with the BS/M DE-identified TFs, the previously generated list was first converted to B73 RefGen_v2 (5b.60) from v1 (4a.53), resulting in 885 possible leaf blade TFs that could be directly compared. The cell-specific transcriptomes in this study are from the same material and stages, with the exception of the leaf base, which is not amenable to LM. We find that 20% of the TF transcripts are BS-biased and 14% are M-biased (Fig 7A and Supplementary dataset S5). Transcripts for eight of nine TFs initiate expression in SST stage with M-enhanced expression and switch to BS-enriched in later stages. A significant number of the TFs detected in cell-specific transcriptomes had not been detected in the whole leaf transcriptomes, suggesting that the cell-specific method is more sensitive to low levels of expression.

Several other studies used alternative approaches to identify candidate C_4_-related TFs (Pick *et al.*, 2011; Chang *et al.*, 2012; Wang *et al.*, 2013), enabling comparison for consensus (Fig. 7B and Supplementary dataset S5). Note that these studies all used comparable versions of maize genome annotation. One study (Pick *et al.*, 2011) obtained stage-specific but non-cell-specific transcriptomes from the maize leaf developmental gradient and identified candidate TFs with the same developmental dynamic as one of three key C_4_ pathway enzymes. That study identified TFs based on co-expression with the NADP-malic enzyme; we find four are BS-enhanced, although none of the other alternative methods identified them as C_4_-related candidates (Fig. 7B).

Another cell-specific transcriptome study (Chang *et al.*, 2012), employing mechanical isolation of BS and M cells from more mature leaves of maize cultivar White Crystal, identified cell-specific TFs using a rigorous criterion for specificity (Ri>.99). 40% were also detected in our DE lists, which accept a greater window of differential expression in BS and M cells. Our analysis identified comparable numbers of BS and M-specific candidate TFs, whereas the study of Chang *et al.* (2012) identified many fewer M-specific candidates. For five (2%) of the genes, we detected significant transcript in the opposite cell type with a changing developmental dynamic (i.e. our study-GRMZM2G336533: log_2_ BS/M SST=0.8, maturing=0.9, mature=–1.1; Chang *et al.* 2012 study; M cell-specific).

A third study (Wang *et al.*, 2013) employed the comparison of transcriptomes of leaf primordia and husk leaves to identify genes active at early times establishing the C_4_ Kranz features, which are diminished or lacking in husk leaves. Although comparing different stages and materials, the results of this study overlapped with our results to a remarkable extent. Our DE data identified 46 TFs of the 116 C_4_-related candidates found by this method (40%; 26 BS-specific, 20 M-specific). Of the remaining 70, we measure non-cell-specific expression of 58 (83%) in the leaf blade and leaf-base expression of an additional 8 (Li *et al.*, 2010). We find that most of the 46 cell-specific TFs are expressed at a high level at the SST stage, suggesting that they may be dedicated to late C_4_-related development, such as refinement of BS-M cell differentiation and barriers.

The subset of TFs that were identified by all three strategies was detected with the same cell-specificity by both mechanical cell isolation and microdissection. 14 BS-specific and two M-specific TFs were identified in three studies and 178 were identified in at least two studies. The consensus of these studies provides support for a considerable number of candidate TFs that might be profitably investigated further for specific roles in C_4_ development and function.

## Discussion

### Cell-specific transcriptomes from successive developmental stages provide novel tools

We show that cell-specific transcriptomes from BS and M cells at successive developmental stages can augment the insights into C_4_-related processes that can be gained from BS/M studies of a single mature stage or from multiple stages of whole leaves. First, our studies resolve the early BS- and M-specificity of transcripts for C_4_-related processes, before the sink–source transition and before significant illumination. The early expression of C_4_-related genes was observed previously in whole leaves (Li *et al.*, 2010; Pick *et al.*, 2011); our data show that cell-specificity is in place from these earliest stages. Second, these transcriptomes enable the assignment of roles to C_4_-related gene family members primarily expressed at an early developmental stage, the matching of candidate interaction partners that are active early, and the identification of C_4_-related transcription factors, all of which may be absent or difficult to detect in later mature stages. Third, the availability of multiple stages from the same leaves enables the profiling of dynamic cell-specificity for individual genes, some of which are present or cell-specific only in a single stage. Finally, the transcriptome stages reveal a succession of cell-specific activities and roles not previously viewed by other studies, as described below.

### Evidence of stress-related transcription in Kranz cell in mature (tip) stage

We observed several stress-related genes among the highest expressed genes in mature stage BS and M cells. Mature M cells accumulate transcripts from four acireductone dioxygenases (ARD), which have been associated with methionine salvage in *Arabidopsis*. In the ARD-dependent Yang Cycle (Yang and Hoffman, 1984), *S*-adenosyl methionine (SAM) is converted to 5-methyl-thioadenosine (MTA). There are four *ARD* genes in *Arabidopsis*, three of which (*AtARD1, 2, 3*) are expressed in phloem cells along with other identified genes in the cycle (Pommerrenig *et al.*, 2011). *AtARD4* is expressed in the phloem and BS cells.

In maize, our transcriptome data suggest that the entire methionine cycle may function in cooperating BS and M cells; data is lacking for phloem. We observe M-enhanced expression in mature stage of four *ZmARD* genes, all homologous to *AtARD4*. For the single maize gene (GRMZM2G165998) homologous to *AtARD1, 2* and *3*, we observe strong BS-enhanced expression at all stages, consistent with detection of the protein in BS cells (Sun *et al.*, 2009). For methionine salvage steps from MTA to SAM, the homologous maize genes are either BS-enhanced or equal in both cell types. However, the two relevant activities for MTA steps in maize—an *S*-adenosylmethionine decarboxylase (GRMZM2G154397) and an arginine decarboxylase (GRMZM2G374302)—seem to be M-enhanced. Both steps generate CO_2_ and lead to putrescine, which is associated with stress tolerance and regulation of photosynthesis (Kusano *et al.*, 2008; Ioannidis *et al.*, 2012). Polyamines also protect against oxidative damage (Rhee *et al.*, 2007).

Transcripts for several additional stress-related activities are notably cell-specific and abundant at the mature tip. Among these are those for trehalose metabolism (BS-specific and M-specific members), hexokinase signalling (BS-specific), AKIN10 homologues (M-specific at SST, BS-specific at maturing/mature; Fragoso *et al.*, 2009), and a variety of genes related to reactive oxygen species.

The presence of stress-related transcripts is unlikely to be an artefact of experimental conditions that cause leaf tips to dehydrate or senesce prematurely, as they are observed in many independent studies, with plants grown under varying conditions (Li *et al.*, 2010; Pick *et al.*, 2011; Chang *et al.*, 2012). Instead, the encoded activities are likely to belong to a battery of developmental mechanisms that address the potentially damaging by-products of peak photosynthetic and assimilatory activities.

### Use of homeologous genes in maize for C_4_-related processes

The filtered lists contain many pairs of homologous genes. These genes may be homeologues (syn: ohnologues, syntenic paralogues) resulting from an ancient whole genome duplication in the maize lineage (Schnable *et al.*, 2009). The maize whole genome duplication has been dated to 5–12 million years ago (Swigonova *et al.*, 2004) and is shared by the sister genus *Tripsacum*, but not by the core *Andropogoneae* (Bomblies and Doebley, 2005), placing the whole genome duplication shortly after the origin of C_4_ photosynthesis (Aliscioni *et al.*, 2012). Previous work in the crucifers has demonstrated that retention of whole genome duplicates may act as a way for plants to increase flux through whole metabolic pathways (Bekaert *et al.*, 2011), a trait likely under high levels of selection following the transition to a new photosynthetic method. A second model predicts that gene pairs descended from an ancestral gene expressed in both the M and BS might subfunctionalize (Force *et al.*, 1999) with one gene copy specializing in M expression/function and the other in BS expression/function.

In the case of the *TIM* family, the model of increasing metabolic capacity is more consistent with our observations than the subfunctionalization model. The *TIM* family contains two pairs of homeologous genes (four total genes). Each gene pair shares a conserved pattern of expression, with one pair of genes expressed preferentially in the M and the other expressed preferentially in the BS. Another example is the two sets of transcription factors identified in three studies (this study; Chang *et al.*, 2012; Wang *et al.*, 2013). Only one pair (Dof-like) are homeologues and they have very similar expression in this dataset. About a quarter of the closely examined genes (100) that were found in various filtered DE lists were homeologues and were expressed in a similar pattern. This observation suggests that homeologue retention may be an active mechanism to increase protein levels of some activities for C_4_ cycle efficiency and should be investigated more thoroughly.

### Comparison of transcriptome data from alternative isolation methods for BS and M cells

Studies of BS and M cells from maize and other C_4_ species have employed a variety of methods to distinguish the contributions of the two cell types, sometimes with varying results, as described in the Results. The LM method we employed has the advantage that M and BS cells can be isolated from the same preparation, using the same technique. As has been suggested, some level of cross-contamination of adjacent cell types is probably unavoidable, influencing the absolute value of BS-M differences, but not the detection of significant differences. In this study, we evaluated the entire class of differentially expressed genes, from ratios of expression near 1 to absolutely cell-specific, with >85% with a ratio greater than 2. For individual genes, we find mostly good correlation with the BS-M distribution ratios found in proteomic studies of the same source material (Friso *et al.*, 2010; Majeran *et al.*, 2010), with the caveat that the abundance of a significant number of proteins differs from their relative transcript abundance. Our transcriptome data for BS cells is generally consistent with mature stage BS transcriptome data produced in a recent study employing mechanical cell separation (Chang *et al.*, 2012). However, our data for M cells was less consistent with the M cell transcriptome data from that study, which employed a protoplasting method to isolate M cells. Despite the somewhat different values produced by the alternative methods, genes and patterns identified independently by multiple methods are likely to be the strongest candidates for involvement in C_4_-related and other cell-specific pathways.

## Supplementary data

Supplementary data are available on *JXB* online.

Dataset S1. All BS and M DE genes.

Dataset S2. DE genes for C_4_ and other genes mentioned in text.

Dataset S3. The top 100 most highly expressed DE genes in each section.

Dataset S4. DE genes expressed in only one section.

Dataset S5. Transcription factor identification comparison.

Figure S1. Heat maps of log_2_BS/M for metabolic pathways related to C_4_.

Supplementary Data

## Abbreviations:

BS: bundle sheath
DE: differentially expressed
LM: laser microdissection
M: mesophyll
PPDP: plant proteome database (http://ppdb.tc.cornell.edu/)
SST: sink–source transition.
