# Supplementary material for: Developmental dynamics of Kranz cell transcriptional specificity in maize leaf reveals early onset of C4-related processes
Source: J Exp Bot. 2014 Apr 30;65(13):3543–55. doi: 10.1093/jxb/eru152 (PMC4085964; doi:10.1093/jxb/eru152)
Supplement: Supplementary Data [file supp_65_13_3543__index.html]

Developmental dynamics of Kranz cell transcriptional specificity in maize leaf reveals early onset of C4-related processes — Developmental dynamics of Kranz cell transcriptional specificity in maize leaf reveals early onset of C4-related processes — Supplementary Data 

# Developmental dynamics of Kranz cell transcriptional specificity in maize leaf reveals early onset of C4-related processes

## Supplementary Data

Data files

**Files in this Data Supplement:**

- Supplementary Data - Supplementary Data
- Supplementary Data - Supplementary Data
