# Supplementary material for: Developmental dynamics of Kranz cell transcriptional specificity in maize leaf reveals early onset of C4-related processes
Source: J Exp Bot. 2014 Apr 30;65(13):3543–55. doi: 10.1093/jxb/eru152 (PMC4085964; doi:10.1093/jxb/eru152)
Supplement: Supplementary Data [file supp_eru152_jexbot121491_file002.pdf]

Tausta et al.

SUPPLEMENTAL DATA LEGENDS

Supplementary Dataset S1. All BS and M DE genes.

This sheet is the list of 7,994 genes determined to be DE in BS or M cells for three developmental sections of maize leaf blade. For each gene, Mapman annotations and best rice and *Arabidopsis* homologs are given. The $\log_2$ transformed BS/M expression ratio is found in columns H, I, J followed by the average of the upper quartile-normalized expression values from 2 biological isolations of LM-isolated RNA from either BS or M cells in L-Q. FDR values for each section are in columns S,T,U.

Supplementary Dataset S2. DE gene lists for C4, TIM, GS-PK, Aquaporin, IAA, Methionine salvage.

The dataset contains genes mentioned in the body of the paper. Activity was inferred primarily from Mapman annotation. Included in all are the $\log_2$BS/M values determined in this analysis. For the TIM, GS-PK, Aquaporin, IAA lists the normalized data for each cell type and section and the whole section data from (Li *et al.*, 2010) is also included. Blue shading of values of 1 (2 fold) or higher indicates a BS-biased ratio and green indicates values of -1 (2 fold) or lower and an M-biased ratio. For the methionine salvage pathway-related genes only the tip data is presented in addition to the $\log_2$BS/M values.

Supplementary Dataset S3. The top 100 most highly expressed DE genes in each section.

DE gene lists were filtered for the 100 genes with the highest expression in a cell type at one developmental time and are provided here. The genes that were found in only one section from the top 100 lists are also included.

Supplementary Dataset S4. DE genes expressed in only one section.

The DE list was filtered for genes that were expressed only in one developmental section. Expression in the other 2 sections was restricted to less than 0.2.

Supplementary Dataset S5. Transcription factor identification comparison.

The whole section transcription factor list identified by Li et al., 2010 was converted to B73 RefGen_v2 resulting in a final list of 885 genes which was compared to genes identified as potentially having transcription factor activity from the DE list. Column A contains the mapman annotation for the genes identified in the whole section data listed in column B. Column C shows any matches to BS-biased genes and D any M-biased genes. Note a few genes have annotations in both BS and M columns denoting a switch from one cell type bias to the other over the developmental sections.
The second page compares the BS and M-biased transcription factors with datasets from Wang et al., 2013, Chang et al., 2012 and Pick et al., 2011. Column G in blue denotes the matches with our BS-biased results and column H in green denotes the matches with our M-biased data. Columns D, E, F are the log2BS/M ratios for each of the sections.

Supplemental Figure 1: Metabolism-related Transcripts Remain DE from SST.

Heat maps representing DE gene expression for select C4-related metabolic pathways. Columns represent leaf sections from youngest to oldest left to right and rows represent different genes with the predicted activities indicated on the left. Green signifies an M-biased ratio and blue represents a BS-biased ratio. The color scale represents the log2 BS/M value and is from -3 (green) to +3 (blue) where 0 (white) represents no difference in BS and M values.

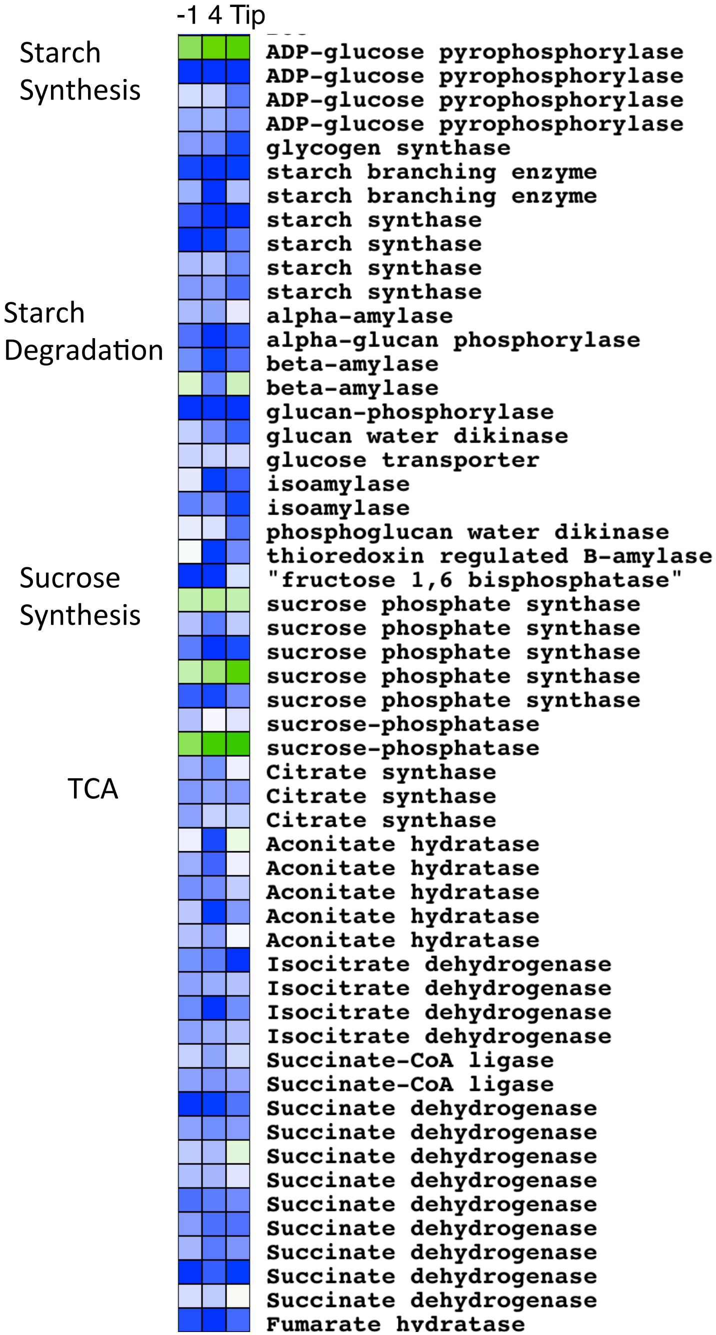
-1 4 Tip
Starch Synthesis
Starch Degradation
Sucrose Synthesis
TCA
ADP-glucose pyrophosphorylase
ADP-glucose pyrophosphorylase
ADP-glucose pyrophosphorylase
ADP-glucose pyrophosphorylase
glycogen synthase
starch branching enzyme
starch branching enzyme
starch synthase
starch synthase
starch synthase
starch synthase
alpha-amylase
alpha-glucan phosphorylase
beta-amylase
beta-amylase
glucan-phosphorylase
glucan water dikinase
glucose transporter
isoamylase
isoamylase
phosphoglucan water dikinase
thioredoxin regulated B-amylase
"fructose 1,6 bisphosphatase"
sucrose phosphate synthase
sucrose phosphate synthase
sucrose phosphate synthase
sucrose phosphate synthase
sucrose phosphate synthase
sucrose-phosphatase
sucrose-phosphatase
Citrate synthase
Citrate synthase
Citrate synthase
Aconitate hydratase
Aconitate hydratase
Aconitate hydratase
Aconitate hydratase
Aconitate hydratase
Isocitrate dehydrogenase
Isocitrate dehydrogenase
Isocitrate dehydrogenase
Isocitrate dehydrogenase
Succinate-CoA ligase
Succinate-CoA ligase
Succinate dehydrogenase
Succinate dehydrogenase
Succinate dehydrogenase
Succinate dehydrogenase
Succinate dehydrogenase
Succinate dehydrogenase
Succinate dehydrogenase
Succinate dehydrogenase
Succinate dehydrogenase
Fumarate hydratase

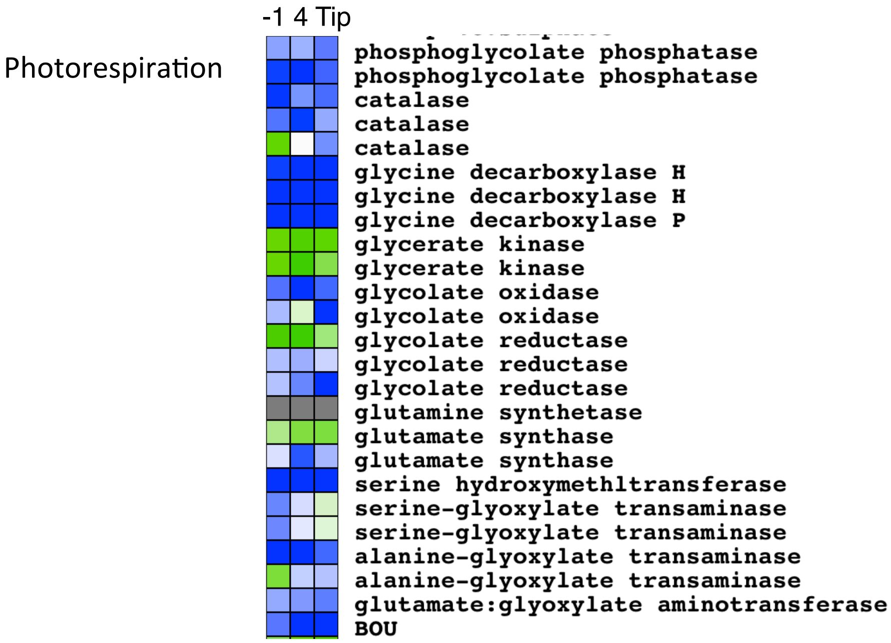
Photorespiration
-1 4 Tip
phosphoglycolate phosphatase
phosphoglycolate phosphatase
catalase
catalase
catalase
glycine decarboxylase H
glycine decarboxylase H
glycine decarboxylase P
glycerate kinase
glycerate kinase
glycolate oxidase
glycolate oxidase
glycolate reductase
glycolate reductase
glycolate reductase
glutamine synthetase
glutamate synthase
glutamate synthase
serine hydroxymethltransferase
serine-glyoxylate transaminase
serine-glyoxylate transaminase
alanine-glyoxylate transaminase
alanine-glyoxylate transaminase
glutamate:glyoxylate aminotransferase
BOU

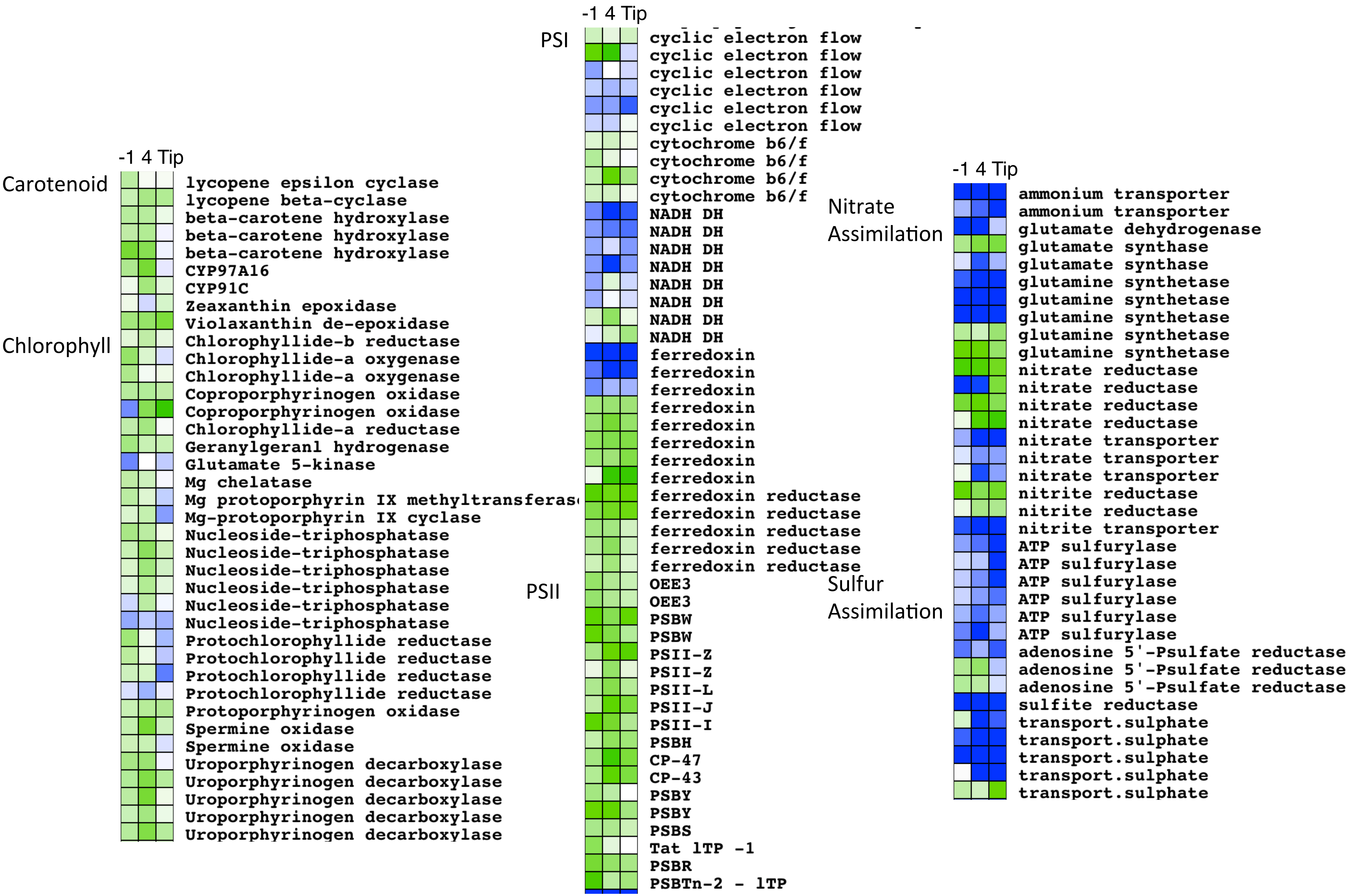

-1 4 Tip
Carotenoid
lycopene epsilon cyclase
lycopene beta-cyclase
beta-carotene hydroxylase
beta-carotene hydroxylase
beta-carotene hydroxylase
CYP97A16
CYP91C
Zeaxanthin epoxidase
Violaxanthin de-epoxidase
Chlorophyll
Chlorophyllide-b reductase
Chlorophyllide-a oxygenase
Chlorophyllide-a oxygenase
Coproporphyrinogen oxidase
Coproporphyrinogen oxidase
Chlorophyllide-a reductase
Geranylgeranl hydrogenase
Glutamate 5-kinase
Mg chelatase
Mg protoporphyrin IX methyltransferas
Mg-protoporphyrin IX cyclase
Nucleoside-triphosphatase
Nucleoside-triphosphatase
Nucleoside-triphosphatase
Nucleoside-triphosphatase
Nucleoside-triphosphatase
Nucleoside-triphosphatase
Protochlorophyllide reductase
Protochlorophyllide reductase
Protochlorophyllide reductase
Protochlorophyllide reductase
Protoporphyrinogen oxidase
Spermine oxidase
Spermine oxidase
Uroporphyrinogen decarboxylase
Uroporphyrinogen decarboxylase
Uroporphyrinogen decarboxylase
Uroporphyrinogen decarboxylase
Uroporphyrinogen decarboxylase
-1 4 Tip
PSI
cyclic electron flow
cyclic electron flow
cyclic electron flow
cyclic electron flow
cyclic electron flow
cyclic electron flow
cytochrome b6/f
cytochrome b6/f
cytochrome b6/f
cytochrome b6/f
NADH DH
NADH DH
NADH DH
NADH DH
NADH DH
NADH DH
NADH DH
NADH DH
ferredoxin
ferredoxin
ferredoxin
ferredoxin
ferredoxin
ferredoxin
ferredoxin
ferredoxin
ferredoxin reductase
ferredoxin reductase
ferredoxin reductase
ferredoxin reductase
ferredoxin reductase
PSII
OEE3
OEE3
PSBW
PSBW
PSII-Z
PSII-Z
PSII-L
PSII-J
PSII-I
PSBH
CP-47
CP-43
PSBY
PSBY
PSBS
Tat lTP -1
PSBR
PSBTn-2 - lTP
-1 4 Tip
Nitrate Assimilation
ammonium transporter
ammonium transporter
glutamate dehydrogenase
glutamate synthase
glutamate synthase
glutamine synthetase
glutamine synthetase
glutamine synthetase
glutamine synthetase
glutamine synthetase
nitrate reductase
nitrate reductase
nitrate reductase
nitrate reductase
nitrate transporter
nitrate transporter
nitrate transporter
nitrite reductase
nitrite reductase
nitrite transporter
Sulfur Assimilation
ATP sulfurylase
ATP sulfurylase
ATP sulfurylase
ATP sulfurylase
ATP sulfurylase
ATP sulfurylase
adenosine 5'-Psulfate reductase
adenosine 5'-Psulfate reductase
adenosine 5'-Psulfate reductase
sulfite reductase
transport.sulphate
transport.sulphate
transport.sulphate
transport.sulphate
transport.sulphate
